# Promising Role of the *Scutellaria baicalensis* Root Hydroxyflavone–Baicalein in the Prevention and Treatment of Human Diseases

**DOI:** 10.3390/ijms24054732

**Published:** 2023-03-01

**Authors:** Marcelina Chmiel, Monika Stompor-Gorący

**Affiliations:** Department of Human Pathophysiology, Institute of Medical Sciences, University of Rzeszów, Kopisto 2a, 35-315 Rzeszow, Poland

**Keywords:** root antioxidants, natural polyphenols, baicalein, pro-health effect, *Scutellaria baicalensis*

## Abstract

Plant roots, due to a high content of natural antioxidants for many years, have been used in herbal medicine. It has been documented that the extract of Baikal skullcap (*Scutellaria baicalensis*) has hepatoprotective, calming, antiallergic, and anti-inflammatory properties. Flavonoid compounds found in the extract, including baicalein, have strong antiradical activity, which improves overall health and increases feelings of well-being. Plant-derived bioactive compounds with antioxidant activity have for a long time been used as an alternative source of medicines to treat oxidative stress-related diseases. In this review, we summarized the latest reports on one of the most important aglycones with respect to the pharmacological activity and high content in Baikal skullcap, which is 5,6,7-trihydroxyflavone (baicalein).

## 1. Introduction

**Figure 1:**
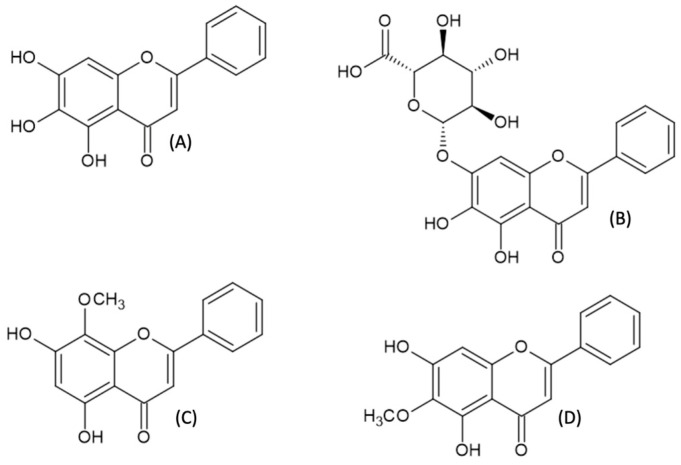
Structures of selected compounds extracted from *Scutellaria baicalensis;* baicalein (**A**), baicalin (**B**), wogonin (**C**), and oraoxilin A (**D**).

Natural flavones with free hydroxyl groups attached to the aromatic A-ring belong to a group of plant polyphenols that are commonly found in nature [1]. They are found in fruits, vegetables, and herbs; therefore, they are a part of our daily diet. After consumption, the compounds are oxidized in the human organism by the cytochrome P450 pathway and by liver microsomal enzymes, which leads to changes in their structures and pharmacological properties [2]. According to the most recent research, these compounds demonstrate a wide spectrum of biological activities, such as antihypertensive [3], proapoptotic [4], inducing hypertrophy of skeletal muscles [5], antiallergic [6], anti-inflammatory, antioxidative, antimicrobial [7], and anti-tumorigenic ones [8]. The substances that are the most promising for potential therapeutic use are subjected to clinical trials [9,10]. *Scutellaria baicalensis* belongs to one of the most important plants in Chinese medicine and has been used for centuries. Compounds such as baicalein, baicalin, wogonin, and oroxylin A (Figure 1) are the main components isolated from the roots of *S. baicalensis*, and their wide application in the treatment of various diseases has been reported [11,12,13]. Moreover, fruits, root barks, and leaves of *S. baicalensis* are abundant in baicalein, with the amount ranging from 12, 54 to 37, 43 mg g^−1^ [14,15]. In the recent study on the chemical composition of dandelion (*Taraxacum mongolicum*), the plant well known for its therapeutic properties, baicalein was found as one of the ingredients, along with newly identified antioxidants, e.g., hesperetin-5-O-β-rhamnoglucoside [16]. However, the content of baicalein in dandelion is much lower than in Baikal skullcap root. Additionally, baicalein was obtained by enzymatic conversion of pinocembrin using recombinant flavone synthase I from Daucus carota (DcFNS I) [17].

Baicalein has been found to exhibit multiple pharmacological properties, including neuroprotective, hepatoprotective, antiviral, and anti-asthmatic ones [18,19,20,21,22]. According to the newest research results, baicalein can reduce myocardial injury by suppressing harmful changes caused by lipid peroxidation in cardiomyocytes [23,24]. Currently, the metabolism of baicalein in humans is under investigation. An initial study has shown that baicalein administered orally is metabolized in almost 95%, and only slightly more than 5% of the compound remains unchanged in the circulatory system. The most abundant metabolites of baicalein were determined to be baicalein-7-*O*-sulfate and baicalein-6-*O*-glucuronide-7-*O*-glucuronide [25].

However, little information is available so far on possible unwanted side effects of baicalein in humans and effective ways of its delivery. We also do not know what safe therapeutic doses of baicalein are.

To support further development of the research in this field, the objective of our work was to summarize the results of current studies on 5,6,7-trihydroxyflavone (baicalein), including its presence in diet, pro-health properties, and interactions with some drugs.

## 2. Biological Activity

### 2.1. Anticancer Activity of Baicalein and Its Derivatives

With the multiple therapeutic benefits of baicalein, its anticancer properties against a broad panel of human cancer cell lines both in vitro and in vivo were documented [26]. It acts through the induction of apoptosis [27], induction of cell-cycle arrest [28], inhibition of proliferation via different signaling pathways such as miR-7/FAK/AKT [29], Akt/mTOR and Nrf2/Keap 1 [30], downregulation of Notch 1/hairy and enhancer of split (Hes) [31], modulation of the activity of Akt/mammalian target of rapamycin (mTOR) pathway [32], and regulation of the Src/inhibitor of differentiation 1 (Id1) pathway [33]. It was also demonstrated that baicalein was able to inhibit the phosphorylation of extracellular-signal-regulated kinase (ERK) and matrix metalloproteinases (MMPs) [34,35]. Moreover, baicalein inhibited cancer-cell migration through the suppression of Wnt/β-catenin and mitogen-activated protein kinase (MAPK) signaling pathways [34]. In line with this, numerous studies showed that baicalein-induced autophagy through the modulation of reactive oxygen species (ROS), signaling and downregulation of vacuolar protein sorting 34 (Vps34), autophagy-related (Atg)5, Atg7, and beclin 1 [36].

Antitumor properties of baicalein were proven for various malignancies (Table 1), including cholangiocarcinoma (HUH28, TFK1, HUCCT1, QBC939, and MZ-Cha-1) [37], gastric cancer (SGC-7901, SGC-7901/DDP, MGC-803, and HGC-27) [30], colorectal cancer (HT-29, HCT-116, SW480, and SW620) [38], multiple myeloma (RPMI 8226) [39], hepatocellular carcinoma (BEL-7402 and BEL-7402/5-FU) [40], breast cancer (MCF-7) [41], osteosarcoma (143 B, MG63, and U2OS) [42], glioma (U251MG) [43], nasopharyngeal cancer (CNE1 and CNE2) [32], and cervical cancer (C33A) [44]. According to the Bonham et al. study [45], oral administration of 20 mg kg^−1^ of baicalein inhibits the growth of prostate cancer xenografts in nude mice by approximately 55%.

Although rapid progress in current cancer treatments has been made over the last years, there are still ongoing studies aiming to find therapeutic agents and their derivatives that would be more potent than the parent compounds.

Previous studies proved the anticancer activity of baicalein derivatives [46]. Baicalein was modified by derivatization of the C6-OH group and by introducing a nitrogen-containing hydrophilic heterocyclic ring to C7-OH via the length of the 3- or 4-carbon chain [47]. The most potent compound with a pyrrolidine ring showed the highest antiproliferative activity against HepG2, A549, and BCG-823 cancer cells, with IC_50_ values of 2.0 μM, 0.8 μM, and 3.2 μM, respectively. Other studies [48] also demonstrated that 7-OBn-6-O substituted baicalein with the piperazine acetamide group at the 6-position has a significant anticancer effect and inhibits the growth of human lung cancer A549 (IC_50_ 4.73 μM). Additionally, numerous studies demonstrated valuable pharmacological properties of the methylated metabolite of baicalein—Oroxilin A, which inhibited the activity of the CYP1B1 mediator, which is responsible for the progression of human breast cancer (IC_50_ 0.0146 and 2.27 μM for oroxilin A and baicalein, respectively) [49,50,51].

### 2.2. Synergistic Effect of Baicalein with Anticancer Agents

Over the past years, naturally occurring compounds with antineoplastic properties took a lot of attention in cancer treatment due to their efficiency and minimal toxicity [52]. Combination or synergistic chemopreventive therapies have been highlighted in order to achieve selectivity of action and the least side effects.

Very successful was the combination of baicalein (0.2 μM) and resveratrol (0.1 μM), which proved efficacious with enhanced synergistic antioxidant effect on human skin fibroblasts (HSF) [53]. Moreover, a combined treatment with an ERK inhibitor (U0126) and baicalein against colorectal carcinoma (CRC) led to the synergistic reduction of MMP-2/9 expression, which is responsible for the anti-metastatic effect in CRC cells [35]. Other studies provided evidence that baicalein, in combination with 10hydroxycamptothecin (HCPT), exerts a significant anticancer effect by triggering DNA damage through targeting topoisomerase 1 (Topo 1) to up-regulate p53 protein, which is a tumor suppressor [54]. There are many reports on the successful treatment of human pancreatic cancer using baicalein combined with gemcitabine or docetaxel [55]. This combination caused a strong suppression of migration of the pancreatic cancer cells and induced their apoptosis via the caspase-3/PARP signaling pathway. Moreover, in another in vivo study, gemcitabine and baicalein were prepared as prodrug-based targeted nanostructure lipid carriers (BCL NLCs). The obtained nanomedicines showed significant tumor growth inhibitory activity in the murine pancreatic cancer model [56]. Similarly, baicalein has been found to enhance the anticancer action of docetaxel in thyroid cancer [57], doxorubicin in breast cancer [58], taxol in ovarian cancer [59], paclitaxel in lung cancer [60], and silymarin in human hepatoma [61].

The antiviral potential of baicalein was observed for many viruses, including dengue virus [62], SARS-CoV-2 [63], influenza [64], and Epstein–Barr virus (EBV) [65].

Recently, the group of Zhang [66] demonstrated that baicalein triazole derivatives prevented respiratory tract infection in RSV-induced human lung cells. The compounds with substituents in the *ortho*-position, containing fluoro, trifluoromethyl, nitril, and bromo groups, enhanced RIG-I and IFN-β1 gene expression, which play significant roles in combating viral infections. Moreover, all the compounds inhibited the secretory activity of interleukins and reduced nitric oxide and malondialdehyde production, showing antioxidant activity. In addition, the effect of baicalein on the inhibition of proinflammatory cytokines production was described against infectious bursal disease virus in embryonic eggs [67].

According to Luo et al. [68], replication of herpes simplex virus type 1 (HSV-1) was inhibited by baicalein treatment due to suppression of ICP27, ICP8, and GB proteins in HaCat cells in all stages of infection. Therefore, dual mechanisms were involved in its antivirus action, impediment of NF-κB activation via inhibiting IKK-B and IκB-α phosphorylation and inactivation of free viral particles in a dose-dependent manner (EC_50_ 3.64 mol L^−1^).

Baicalein may potentially be developed as a novel antiviral and anticancer drug, which can be administered alone or combined with commonly used chemotherapeutics, thus improving the treatment of cancer in the future.

**Table 1:** In vitro studies demonstrating half maximal inhibitory concentration values of baicalein against several types of malignancies.

Cell Lines	Type of Cancer	Assay	Time	Dose, IC_50_ [μM]	Ref.
QBC939	Cholangiocarcinoma	CCK-8	72 h	32.73	[37]
MGC-803	Gastric cancer	MTT	48 h	85.70	[30]
HT-29	Colorectal cancer	MTS	72 h	40	[38]
RPMI 8226	Myeloma	MTT	24 h	168.5	[39]
BEL-7402	Hepatocellular carcinoma	MTT	48 h	54.96	[40]
MCF-7	Breast cancer	MTT	72 h	CC_50_ = 56.46 μM	[41]
U2OS	Osteosarcoma	MTT	48 h	53	[42]
CNE1	Nasopharyngeal cancer	MTT	144 h	20.95	[32]
C33A	Cervical cancer	MTT	96 h	200	[44]

### 2.3. Antimicrobial Activity and Biofilm Formation

Various biological effects of baicalein have been reported, including antimicrobial and antifungal ones [69,70,71]. According to the study by Jang [72], baicalein exhibited antimicrobial activity against cariogenic bacteria and periodontal pathogenic bacteria (MICs 80–320 μg mL^−1^; MBCs 160–640 μg mL^−1^), but the effect was lower compared to ampicillin (MICs 0.25–0.5 μg mL^−1^; MBCs 32–64 μg mL^−1^). Additionally, baicalein showed antibacterial action towards *Staphylococcus aureus* with minimal inhibitory concentration (MIC) of 256 μg mL^−1^ [73]. Moreover, baicalein at a dose of 16 μg mL^−1^ synergistically restored the antibacterial actions of ciprofloxacin against Gram-positive bacteria [74]. In addition, the bacteriostatic effect of baicalein was also shown in combination with baicalein-hydroxypropyl-β-cyclodextrin inclusion complex into polyvinyl alcohol nanofibers (PVA-Ba-IC-NF) against Gram-negative bacterium *Escherichia coli* [75]. The latest studies confirmed the antibacterial activity of baicalein against *Staphylococcus epidermidis* with a MIC value of 34 μg mL^−1^ (corresponds to 126 μmol L^−1^) [76]. What is more, in the same report antimicrobial action of baicalein adsorbed on the hydroxyapatite layer is described. Bacterial growth was significantly reduced (by one order of magnitude) on the baicalein-HAp particles compared to pure hydroxyapatite.

Another feature of baicalein is its antifungal activity. The group of Serpa [77] demonstrated inhibitory effect of baicalein against several *Candida* strains (*C. albicans*, *C. tropicalis*, *C. parapsilosis*) with MIC_50_ values ranging from 13 to 104 μg mL^−1^. Moreover, all tested species exposed to baicalein showed a high loss of viability through ROS accumulation. For *Candida krusei* isolates, baicalein exhibited in vitro antifungal activity with a MIC value of 2.7 μg mL^−1^ [78]. Antifungal activity has also been tested on *Aspergillus fumigatus,* where baicalein at the concentration of 0.25 mM inhibited the growth of the fungus by 90% [79]. Additionally, baicalein, at the same amount, remarkably decreased the number of conidia adhering to the surface of human corneal epithelial cells (HCECs). Furthermore, in the microbroth dilution method, the antifungal action of baicalein was observed against human pathogenic fungi *Trichophyton rubrum* and *Trichophyton mentagrophytes* [80]. Pronounced growth inhibition was detected at MICs doses of 0.12 mM and 0.06 mM for *T. rubrum* and *T. mentagrophytes*, respectively.

Further studies were conducted to evaluate synergism between baicalein and other antifungal compounds. A combination of baicalein and berberine hydrochloride showed a strong inhibitory effect on the growth of *Candida albicans*, with a fractional inhibitory concentration index (FICI) of 0.5. Moreover, the combinations of baicalein-quercetin and baicalein-fluctonazole also demonstrated synergistic interactions against *C. albicans* with FICI values of 0.37 and 0.32, respectively [81].

Biofilm formation is a common strategy of bacteria in response to environmental stress [82]. The development of a biofilm intensifies the capacity of bacteria to evade antibiotics by blocking their penetration through the bacterial biofilm layers. This may be very dangerous for patients.

It was confirmed that baicalein, in combination with linezolid, had the potential to decrease biofilm formation by over 50% [83]. An in vivo study [73] showed that baicalein reduced cell attachment and was able to eradicate 7-day biofilms of *Staphylococcus aureus* in a dose-dependent manner. Furthermore, treatment with baicalein and vancomycin remarkably reduced the number of bacteria on the carrier. Other studies have been carried out to investigate the efficacy of baicalein-coated gold nanoparticles (BCL-AuNPs) against biofilm formation by *Pseudomonas aeruginosa* [84]. It was indicated that BCL-AuNPs significantly attenuated bacterial biofilm formation (by approximately 60%). Recent reports revealed a good anti-biofilm potential of baicalein against avian pathogenic *Escherichia coli* (APEC) [85]. In the range from 12.5 to 50 μg mL^−1,^ baicalein significantly reduced the biofilm formation and adhesion capacity, and this activity was associated with curli fimbria genes—*csgA* and *csgB*.

These results suggest that baicalein, in association with various antibiotics, is an effective way to overcome the mechanisms of bacterial resistance and may be used in antifungal therapy.

### 2.4. Antioxidant Activity

Among other significant biological activities of baicalein, it is worth noting that it has antioxidant and anti-inflammatory properties [81,86]. Oxidative stress plays a major role in the pathogenesis of chronic diseases such as respiratory and cardiovascular diseases, diabetes, and neurotic disorders. These activities of baicalein are mainly due to its ability to scavenge reactive oxygen species (ROS) via various mechanisms, in particular by attenuation of the activity of NF-κB [87] and suppression of the expression of various inflammatory cytokines and enzymes (e.g., COX, TNF, IL, NO) [88].

Ma and co-workers [89] reported that baicalein successfully inhibited H_2_O_2_-induced cytotoxicity and apoptosis in human vitiligo melanocytes (PIG3V) by abolishing Nrf2 knockdown. On top of that, baicalein promoted the expression of Nrf2 nucleus translocation and its target gene, oxygenase-1 (HO-1). The same conclusions were drawn by Lee [90] in an in vivo study on Chinese hamster lung fibroblasts (V79-4). Because of its chemopreventive activity, baicalein improves antioxidant status in lung carcinogenesis by reducing DNA damage in lung tissue and restoring the elevated glycoprotein level to normality [91]. The ability of baicalein to indirectly inhibit •OH radical production was observed in bone marrow-derived mesenchymal stem cells through the Fe^2+^-chelation pathway [92].

### 2.5. Antidiabetic Activity of Baicalein

Diabetes is a chronic metabolic disease characterized by insufficiency in insulin secretion from pancreatic β-cells, which leads to the accumulation of glucose in plasma and eventually to hyperglycemia [93]. Type 1 diabetes (T1D) is an autoimmune disorder resulting from the destruction of the insulin-making cells in the pancreas, while type 2 diabetes (T2D) is due to insulin resistance, which is the case when cells do not respond properly to insulin [94]. A decrease in insulin action is accompanied by the upregulation of insulin secretion and vice versa. Dysregulation of these metabolic pathways is a main factor leading to both T1D and T2D [95]. In consequence, an agent capable of improving the function of the pancreatic *β*-cells would be potent for the treatment of diabetes.

A diet containing baicalein (250 and 500 mg/kg/day) administered to mice with non-genetic type 2 diabetes fed with a high-fat diet (HFD) led to a significant amelioration of glucose tolerance, hyperglycemia, and blood insulin levels compared to the control group [96]. These effects of baicalein are associated with the improvement of islet *β*-cell survival and mass. In another animal model study, oral administration of baicalein (400 mg/kg/day) to HFD-fed mice improved the severity of obesity, insulin resistance, inflammation, hyperglycemia, and hyperlipidemia in diabetic mice [97]. Supplementation with baicalein revealed that inhibition of inflammation and insulin resistance works through activation of AMPK. Additionally, baicalein (90 mg mL^−1^) pretreatment to primary culture of hepatocytes from the liver of wild-type mice and AMPKa2-stimulated mice in glucose solution (25 mM) showed noteworthy inhibition of MAPKs signaling pathway by downregulation of ERK and p38 phosphorylation in a wild-type hepatocyte culture. However, it was no such effect in AMPKa2-stimulated hepatocytes [98].

In another study carried out by the group of Fu et al. [99], the treatment of insulin-secreting pancreatic INS382/13 cells and human islets cultured under hyperlipidemic conditions with baicalein at a dose of 5 mM significantly elevated glucose-stimulated insulin secretion (GSIS) and promoted the viability of the insulin-secreting cells and the islets.

These findings demonstrate that baicalein may serve as a natural antidiabetic agent that directly modulates pancreatic *β*-cell function and significantly improves metabolic syndrome disorders by blocking the AMPKa2-mediated MAPKs signaling pathway.

### 2.6. Baicalein’s Activity in Respiratory Diseases

Pulmonary fibrosis is a severe lung condition in which collagen is excessively accumulating in the extracellular matrix, leading to respiratory failure [100]. Although the mechanism by which baicalein suppresses the increase in collagen in fibroblasts remains unknown, recent studies suggest that baicalein attenuates mRNA molecules and downregulates connective tissue growth factor (CTGF) in correlation with transforming growth factor β1 (TGF β1) [99].

Long-term supplementation of baicalein (50 and 100 mg/kg/day) to bleomycin-induced pulmonary fibrotic rats notably decreased the severity of pulmonary fibrosis in the baicalein-treated rats [101]. Baicalein demonstrated the antifibrotic effect by lowering levels of miR-21 and downregulating expression of TGF-β1, as well as by reduction of hydroxyproline and alpha-smooth muscle actin (α-SMA) levels in the lung tissue.

The human mast cells (HMCs) are multifunctional tissue-dwelling cells associated with the allergic response. They are capable of regulating inflammation and are involved in host defense and innate immunity [102]. The HMCs display different pharmacological properties depending on their locations within tissues. They express high-affinity receptors for antibodies [103], have the ability to secret inflammatory cytokines (e.g., IL-6 and IL-8) [104], and mediate in antigen-induced inflammation of the respiratory endothelium [105].

In an in vivo assay performed by Hsieh and co-workers [106], baicalein at a dose of 30 μM inhibited the production of cytokines IL-6, IL-8, and MCP-1 from IL-1β and TNF-α-activated culture of human must cells (HMCs). The inhibitory effects are associated with the suppression of NF-κB activation and IκBα phosphorylation and degradation (Table 2).

Consequently, these findings indicate the usefulness of baicalein in the treatment of allergic and asthmatic disorders in humans by regulation of the NF-κB pathway. What is more, baicalein demonstrated a protective effect against acute lung injury (ALI) through direct and selective binding to myeloid differentiation factor 2 (MD2) [107].

### 2.7. Anti-Inflammatory Effect of Baicalein in Food Allergy

Bowel diseases are a group of disorders caused by chronic inflammation of the gastrointestinal tract. It happens through interference or dysfunction of regulatory T cells (Treg), which play a pivotal role in immune homeostasis [108].

In a mouse model study, administration of baicalein at a dose of 20 mg kg^−1^ to mice with ovalbumin-induced food allergy alleviated the symptoms of food allergy and reduced the level of serum IgE and effector T cells by induction of CD4^+^Foxp3^+^T cell differentiation [109]. Food allergic immune response was attenuated by differentiation of Treg cells through the aryl hydrocarbon receptor and by enhancement of intestinal barrier function. In addition, supplementation with baicalein may contribute to the prevention of food allergic disorders and inflammatory bowel diseases.

Additionally, baicalein (10 mg kg^−1^ or 25 mg kg^−1^) was administrated to mice with experimental colitis in combination with curcumin [110]. Co-administration of baicalein and curcumin ameliorated pathological symptoms of colonic inflammation, such as the severity of rectal bleeding and diarrhea, by blocking the expression of downstream enzymes, COX-2, iNOS, and cyclin D1, which are associated with the induction of colitis. The synergistic effect was much stronger than those achieved with each of the compounds alone.

Thus, further research on baicalein potency might be essential to understand its mechanism of action and could be helpful in preventing inflammatory bowel diseases caused by food allergies.

### 2.8. Baicalein’s Activity in Cardiovascular Diseases

**Figure 2:**
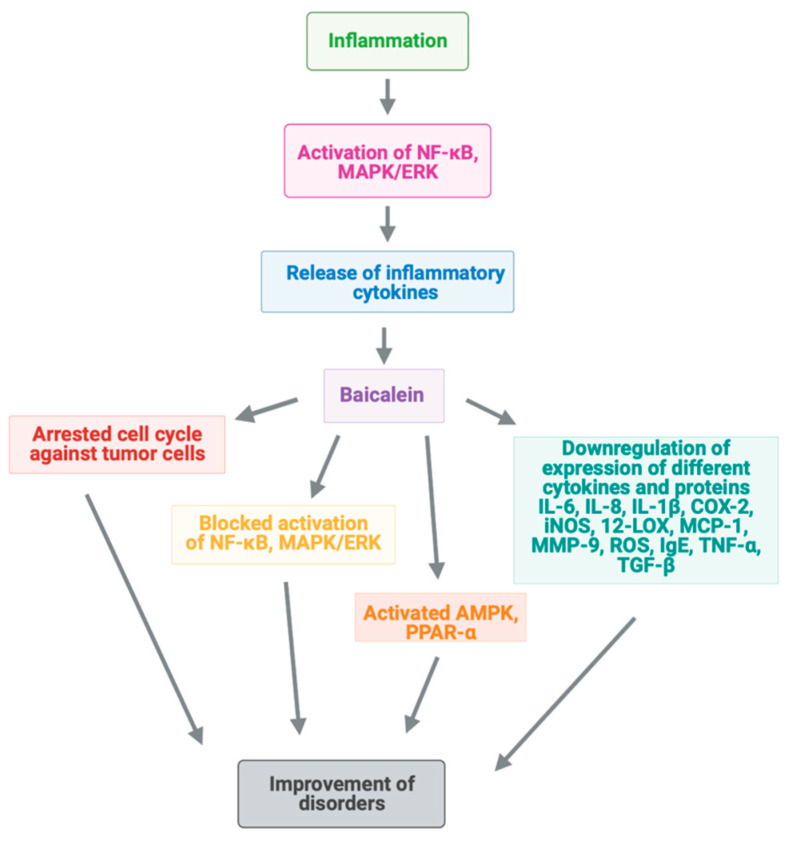
Mechanisms of baicalein action for amendment of inflammation via different signaling pathways.

Cardiac fibrosis occurring after myocardial infarction is frequently the cause of morbidity and mortality [111]. Activation of 12-lipoxygenase (12-LOX) has been shown to promote neuronal death, along with overexpression of MMP-9 (Figure 2) [112]. Additionally, brain natriuretic peptide (BNP) is considered a key marker in heart failure [113].

Oral administration of baicalein (200 mg kg^−1^ per day) for a 12-day period to spontaneously hypertensive rats (SHRs) attenuated myocardial fibrosis by diminution the collagen content in the left ventricle and reduced both systolic blood pressure and plasma BNP level. These happened by suppression of the expression or activity of 12-LOX, pERK, and MMP-9 in cardiac tissue [114]. Therefore, baicalein could be an adequate agent for the treatment of hypertension-related cardiac fibrosis.

Macrophage cholesterol accumulation and foam cell formation are the crucial steps leading to the pathogenesis of atherosclerosis [115]. Under oxidative stress, reactive oxygen species (ROS) produced by vascular cells oxidize LDL to form oxidized low-density lipoproteins (oxLDL). These particles are taken up by activated macrophages through their scavenger receptors. This leads to the cellular accumulation of cholesterol and oxysterols [116].

Most recent studies showed that baicalein suppressed oxLDL-induced cholesterol accumulation by reducing oxLDL uptake through competitive inhibition of the CD36 binding to the epitope structure of oxLDL [117]. Furthermore, this junction (baicalein to CD36 receptor) enhanced the cholesterol efflux through the CD36-Src-JNK-ABCA1 signaling pathway.

Pretreatment of the cultured high glucose-stimulated human umbilical vein endothelial cells (HUVECs) with baicalein at a dose of 10 mM suppressed vascular inflammation due to inhibition of NF-kB activation, attenuation of expression of cell adhesion molecules (CAMs), decreasing cell-cell adhesion/migration and diminishing disruption of endothelial barrier function [118].

Another in vivo study demonstrated that post-treatment with baicalein (20 mg kg^−1^) in LPS-induced septic rats effectively ameliorated cardiovascular dysfunction by improving blood pressure and survival rate through the inhibition of NF-kB activation and reduction of elevated levels of plasma necrosis factor α (TNF-α), iNOS protein, NO and superoxide anions [119]. Moreover, further studies on LPS-induced septic rats showed that supplementation with baicalein (10 mg kg^−1^) improved cardiac contractile function by reducing the oxidative stress and apoptosis through induction of cardiac HO-1 production and reduction of increased levels of iNOS and MCP-1 protein [120]. Other works also revealed that baicalein plays a protective role in myocardial damage [121].

### 2.9. Baicalein in Diet

Insufficiency of nutrients may have a negative impact on human and animal health and lead to the derangement of homeostasis. Naturally derived supplements are desirable in a diet due to their numerous physiological benefits [122].

Supplementation of baicalein (0.1% and 0.3% of initial body weight per day) to koi carp fish (*Cyprus carpio*) significantly increased their weight gain rate, specific growth rate, and spleen index and simultaneously decreased their liver-to-body weight ratio leading to improvement of the growth performance [123].

Additionally, a long-term diet supplemented with baicalein (100 and 200 mg kg^−1^) to broiler chickens demonstrated a noteworthy increase in the body weight and feed conversion ratio of the birds compared to the basal diet [124]. Furthermore, triglycerides and low-density lipoprotein cholesterol were significantly decreased after the intake of baicalein compared to the chickens fed with the control diet. Other studies have been established on the same chicken model, but the animals were fed with baicalein during the early post-hatch stage [125]. These data indicated that breast muscles, as well as subcutaneous and abdominal fat weights, were reduced in chicks fed with 500 mg kg^−1^ of baicalein.

These observations provide evidence for the opposite double-effect of baicalein supplementation, depending on the period of maturation.

### 2.10. Antidepressant Action of Baicalein

Depression is a common mental disorder in which monoamines are the major neurotransmitters [126]. Nearly all compounds that are able to inhibit monoamine reuptake have been proven to be clinically effective antidepressants [127].

In the animal model study, treatment of mice with lipopolysaccharide (LPS) (5 mg kg^−1^) induced depression-like behavior, then administration of baicalein (3 mg kg^−1^) notably diminished the duration of their immobility in behavioral tests, indicating that baicalein can normalize depression-like symptoms [128]. Moreover, upon treatment with baicalein the production of brain-derived neurotrophic factor (BDNF), which is a critical regulator in neuronal survival, decreased significantly. Similar conclusions were drawn by Xiong and co-workers [129].

Earlier reports suggest that baicalein demonstrates antidepressant action through the inhibition of MAO A, the enzyme which plays a crucial role in CNS regulation [130]. The most current studies indicate that baicalein (20 mg kg^−1^) promotes neuronal maturation and rescues neurons from apoptosis via inhibiting activation of the GSK3β/NF-κB/NLRP3 signal pathway in chronic unpredictable mild stress mice (CUMS) [131].

Other studies have been conducted to assess the therapeutic potential of baicalein for the treatment of brain injury caused mostly by an ischemic stroke.

The team of Yang [132] proved that baicalein enhanced neurobehavioral function recovery after ischemia-reperfusion brain injury. The effects of treatment with baicalein, such as suppression of neuronal swelling and restoration of a dense arrangement of neurons, appear to be due to the regulation of microglia/macrophages M_1_/M_2_ transformation. On that basis, the latest reports demonstrate that baicalein attenuates ferroptosis activity through GPX4/ACSL4/ACSL3 axis [133].

These data suggest that baicalein is likely to protect neurons by reducing neuroinflammation, apoptosis, and oxidative stress, and therefore it is an effective agent for neurological dysfunctions.

**Table 2:** Summary of the protective effects of baicalein in various experimental models.

Experimental Model	Mechanism of Action	Effect	Ref.
Mouse	Promoted pancreatic β-cell insulin secretory function	Antidiabetic	[100]
Rat pulmonary fibrosis model	Repressed miR-21 expression	Antifibrotic	[101]
Cell culture of HMC-1 cells	Inhibited IL-6, IL-8, and MCP-1 production	Anti-inflammatory	[106]
Mouse	Decreased level of serum IgE, mMCP-1, Th-1, and Th-17	Antiallergic	[109]
Sodium-induced mouse colitis	Attenuated activity and phosphorylation of IKKβ	Anticancer	[110]
Culture of THP-1 macrophages	Inhibited intracellular cholesterol accumulation	Anti-atherosclerosis	[117]
LPS-induced rats septic shock	Ameliorated increase in hepatic TNF-a, and inhibited iNOS protein expression	Protective effect against endotoxemia	[119]

## 3. Conclusions

Baicalein is one of the most potent antioxidants contained in Baikal skullcap, with significant anti-inflammatory activity. More and more research teams all over the world also confirm its high anticancer potential. Moreover, it was demonstrated that baicalein is beneficial in the treatment of metabolic diseases, e.g., diabetes, cardiovascular diseases (such as hypertension), and respiratory diseases, including allergic and asthmatic ones. There is some evidence that baicalein may help in the treatment of nervous system disorders, including neurological diseases, by preventing destructive changes to neurons caused by, for example, oxidative stress. Baicalein may also be used as a therapeutic agent to inhibit the development of pathogenic microorganisms, such as coagulase-positive *Staphylococcus aureus*, *Enterobacteriaceae,* and yeasts of the genus *Candida*, which are often responsible for nosocomial infections and development of many diseases. The data presented above spur further research on finding optimal ways of baicalein delivery and the methods of its medical use for people. Along with a dynamic increase in the number of patients, especially oncology ones, and the emergence of new human diseases, and because of the acquired drug resistance to therapies used so far, there is a growing need for new initiatives aiming to develop more precise methods of targeted therapies. Ingesting natural antioxidants with food is one of the safer health promotion strategies. Determination of safe therapeutic doses of natural low-molecular-weight polyphenols, including baicalein, may be effective in the treatment of oxidative-stress-related disorders, which play an important role in the pathogenesis of many diseases, including cancers, cardiovascular diseases, and neurodegenerative disorders. Therefore, the next steps of the research should focus on providing evidence that baicalein has no side effects and on the determination of effective physiological concentrations of baicalein based on detailed preclinical studies, followed by clinical trials in people.

## Data Availability

Data is contained within the article.
